# Genetic diversity of toxigenic *Fusarium verticillioides* associated with maize grains, India

**DOI:** 10.1590/1678-4685-GMB-2022-0073

**Published:** 2023-04-07

**Authors:** Vishwambar D. Navale, Amol M. Sawant, Koteswara Rao Vamkudoth

**Affiliations:** 1 CSIR-National Chemical Laboratory, Biochemical Sciences Division, Pune, Maharashtra, India.; 2 Academy of Scientific and Innovative Research (AcSIR), Ghaziabad, India.

**Keywords:** Fusarium verticillioides, diversity, mycotoxin, pathogenicity, food safety

## Abstract

In the present investigation, prevalence, genetic diversity, and mycotoxin producing potential of *Fusarium* species associated with maize grain samples were studied from different geographical regions of India. The highest prevalence of *Fusarium verticillioides* was recorded as 88.52%, followed by *F*. *coffeatum*, *F*. *foetens*, and *F*. *euwallaceae,* 6.55%, 3.27%, and 1.63%, respectively. We isolated 54 strains of *F*. *verticillioides*, and their genetic diversity was studied by inter simple sequence repeats (ISSR). The ISSR fingerprints (AG) 8C and (AG) 8G showed 252 and 368 microsatellite sites in the genome of *F*. *verticillioides* and resulted in 99-100% repeatability and reproducibility. The Simpson (SID) and Shannon (H) indices (0.78 and 2.36) suggest that *F*. *verticillioides* strains exhibit moderate to high diversity. Molecular detection of fumonisin B1 (FB1) biosynthetic genes (*FUM1* and *FUM13*) involved in FB1 production in *F*. *verticillioides* was confirmed by polymerase chain reaction (PCR). Furthermore, 91% of the strains were positive for FB1 production, which was affirmed by liquid chromatography with tandem mass spectrometry (LC-MS-MS). *In-vitro* appurtenance of *F*. *verticillioides* spores exhibited a high to moderate effect on the growth and development of the maize. The current finding demonstrated that most *F*. *verticillioides* strains showed a wide range of genetic diversity with varied toxigenic and pathogenic potentials. In conclusion, for the first time, *F*. *coffeatum*, *F*. *foetens*, and *F*. *euwallaceae* species were reported from maize grain samples in India. They were positive for FB1 and negatively affecting grain quality, which is a major concern in food safety.

## Introduction

Maize (*Zea mays* L.) is a widely distributed cereal crop after wheat and rice, and it is used to make a variety of food products for both human and animal consumption (USDA, 2019). Maize is used for various purposes, such as livestock feed (45%), human food (11%), and industrial purposes (44%) (USDA, 2018). Maize has a high nutritional value, starch (60-68%), proteins (7-15%), fat (5-6%), vitamins (pantothenic acid, folate, vitamin B6, and niacin), and minerals (manganese, phosphorus, magnesium, zinc, and copper), which aid in neuronal cell metabolism and cell renewal. It also has antioxidant activity that protects the cell’s DNA from damage and improves digestion (Bathla et al., 2019). The Food and Agriculture Organization (FAO) predicted that the overall demand for maize would increase to around 300 million tons by 2030 (FAO, 2017). 

*Fusarium* is a ubiquitous plant pathogenic fungus that endangers plant growth and consistently adulterates economically important agricultural produce (Ekwomadu et al., 2018; Balendres et al., 2019). *Fusarium graminearum*, *F*. *culmorum*, *F*. *avenaceum*, *F*. *poae*, *F*. *sporotrichioides*, *F*. *equiseti*, *F*. *verticillioides*, and *F*. *proliferatum* are the predominant species that infect maize, rice, and wheat, etc., and cause vascular wilt, head blight, root rot, stem canker and plant death (Lorenzo et al., 2012; Pasquali et al., 2016; Gabriela et al., 2018;). *Fusarium verticillioides* is a dominant species associated with maize in India (Mohammed et al., 2016), widely spread throughout the world, thus affecting the yield, nutritional value, and quality of the grains (Khosrow, 2017). 

*Fusarium* species contaminate stored grains, fruits, cereals, nuts and produce numerous mycotoxins with diverse chemical structures and biological activities (Askun, 2018; Mohammed and Mohammed-Ameen, 2019; Summerell, 2019). Fumonisins (FUM), zearalenone (ZEA) and trichothecenes (TRI), the most potent mycotoxins produced by *Fusarium* species.are known to cause acute to chronic toxicity in humans and animals (McCormick et al., 2011; Jimenez-Garcia et al., 2018). The exposure of humans and animals to mycotoxin defiled food could result in teratogenicity, estrogenic effects, carcinogenesis, immune suppression, and neurotoxicity (Navale *et al*., 2022a). Fumonisin B1 (FB1) is an inhibitor of ceramidase synthase, an essential enzyme in sphingolipid biosynthesis. FB1, contaminated food leads to detrimental effects in humans and animals. It is hepatotoxic, nephrotoxic, and carcinogenic, and causes esophageal cancer (Chilaka et al., 2017), neural tube defects (NTD), and congenital cerebral defects (Ortiz *et al*., 2015). In addition, the intake of toxins infected food grains by humans and animals could result in low nutrient absorption, retarded growth in infants, malnutrition, immunosuppression, and the gut microbiota (Fernandes et al., 2017). 

*Fusarium* is a diverse fungal genus with over 300 species reported globally (Gary, 2017; Crous et al. 2021). However, species level identification is a challenging task for mycologists and food microbiologists due to closely related species and more than 20 species complexes (Crous *et al*. 2021). The barcoding of the internal transcribed spacer (ITS), translational elongation factor 1α (TEF-1α), RNA polymerase II subunit (RPB2), and β-tubulin (Bt2) could assist in classifying the species within the species complex (Al-Hatmi et al., 2016; Moussa et al., 2017). Furthermore, micro-satellite inter simple sequence repeats (ISSR) is an important marker to study the genetic diversity for identifying highly polymorphic multilocus markers (Wu et al., 2018). Microsatellite fingerprints differentiate closely related species complexity, genetic diversity, genome mapping, and evolutionary lineages (Mohammed et al., 2016; Kozak et al., 2019). 

The food and feed matrices are contaminated with several mycotoxins; hence, it is crucial to identify multiple mycotoxins producing fungi using a simple PCR in a single reaction (Rahman et al., 2020). The European Commission set FUM tolerable limits in raw cereals and grains are 2000-4000 µg/kg (EU, 2006). The FB1 contamination in maize grains has exceeded the tolerable limits in India (Nayaka et al., 2009). However, there is a lack of well-managed initiatives to address mycotoxin problems in India (Seo et al., 2001; Vamkudoth et al., 2016; Nayaka *et al*., 2009). To address such issues, the present study aimed to assess the phylogenetic relationship and genetic diversity of mycotoxigenic *F*. *verticillioides* using ISSR fingerprints. Further, mycotoxin chemotypes were evaluated by precise molecular and liquid chromatography with tandem mass spectrometry (LC-MS-MS) to assure grain quality for the safety of consumers.

## Material and Methods

### Sampling

A total of 140 stored maize samples were collected from the poultry industries of 10 states, including Maharashtra (MH), Rajasthan (RJ), Madhya Pradesh (MP), Andhra Pradesh (AP), Uttar Pradesh (UP), Bihar (BH), Tamilnadu (TN), Karnataka (KA), Jharkhand (JK), and Uttarakhand (UK), India.

### Isolation and identification of *Fusarium* species

The collected maize samples were analyzed for the presence of *Fusarium* by blotter method (ISTA, 1993), serial dilution method (Navale et al., 2022b), and isolates were maintained on Spezieller Nahrstoffarmer agar (SNA) medium (Leslie and Summerell, 2006). *Fusarium* species were identified based on macroscopic observations including color of the colony, morphology, reverse color, and pigment production. Microscopic observations, such as the shape and size of the micro and macro conidia, the arrangement of the conidia, and the growth rate on agar media were used for the taxonomic studies (Leslie and Summerell, 2006). 

## Molecular identification of *Fusarium* species

### Genomic DNA/RNA isolation

The genomic DNA was extracted from *Fusarium* species after they achieved optimum growth at 72 h. About 100 mg of mycelia were ground to powder in liquid nitrogen using mortar and pestle. According to the manufacturer’s instructions, the genomic DNA was extracted using DNeasy Plant Mini Kit (Qiagen, Germany). The concentration and purity of extracted DNA was determined using a Nanodrop Spectrophotometer (Thermo Scientific, USA) and stored at -20 °C. In this study, RNA was extracted using RNeasy Plant Mini Kit (Qiagen, Germany) for the protein coding gene of TEF1-α to study the genetic diversity using IISR fingerprint. For reverse transcriptase PCR, total RNA was transcribed using a high-capacity reverse transcription kit (Thermo Fisher Scientific, USA) and an oligo-dT primer. The first-strand cDNA product was used in the PCR with the TEF1-α gene specific primers EF1 F and, EF2 R (Table S1). Further, the species specificity was confirmed with PCR assay using species-specific primer set Vert 1, and Vert 2 primer set specific to *F*. *verticillioides* (Table S1). PCR was performed in Applied Biosystem Veriti (Thermo Fisher Scientific, USA) with a reaction volume of 20 μL. The amplification mixture consisted of 1 μL of each forward and reversed primers (10 µmol L-1), 1 μL template, 10 μL*Taq* master mix (Jumpstart, Sigma Aldrich, USA), and 8 μL MilliQ water. The PCR program was set as an initial denaturation at 95 ºC for 3 min, followed by 35 cycles of 95 ºC for 30 s, 52 ºC for 30 s, and 72 ºC for 1 min, with a final extension at 72 ºC for 10 min. After successful amplification, the PCR product was purified and sequencing was performed as reported (Sawant et al., 2019).

### Primer designing

Primers were designed for the molecular detection of mycotoxins by targeting their biosynthetic pathway genes such as FUM1 and FUM13 involved in biosynthesis of FB1 using Snap Gene Viewer and analyzed with the help of PRIMER-BLAST and BLASTN tools to eliminate, if any non-specific targets in the PCR assay.

### Molecular detection of FUM gene by PCR assay

The molecular detection of FB1 was carried out by targeting biosynthetic pathway genes FUM1and FUM13 respective to FB1 production. The PCR was carried out in a 20 µL reaction mixture containing 50 ng template, 10 μL*Taq* master mix, 8 μL MilliQ water, 1 μL primers (2 µmol L^-^1) for each gene. The PCR cycling was set as an initial denaturation at 94 °C for 4 min, followed by 35 cycles at 94 °C for 30 sec, annealing at 52 °C for 30 sec and extension at 72 °C for 1 min with a final extension of 72 °C for 10 min. The PCR products were loaded onto 10 g L^-1^ agarose gel electrophoresis and visualized under the gel documentation system.

### Effect of *Fusarium* on maize growth inhibition

*In-vitro*, the effect of *Fusarium* species on seed germination, growth (root and shoot) of maize was determined using the Water-agar (WA) method (Baxter et al., 1994). Maize seeds were surface sterilized with 0.01% HgCl_2_ and soaked in 1×10^6^ spores/mL for 12 h on an orbital shaker. Seeds were soaked in sterile MilliQ water without spore suspension was used as control. Following incubation, seeds were transferred into plant tissue culture bottles (43 mm) containing water agar (2%) under aseptic conditions and incubated for 15 d at 25 °C in 16 h light and 8 h dark conditions. At the end of the incubation period, root and shoot inhibition was measured. Furthermore, seed germination was evaluated by using 7 days grown culture filtrate (CF) of *Fusarium* species (Vamkudoth et al., 2014). On the other hand, 50 ml of CF of all the strains of *Fusarium* were extracted with ethyl acetate (1:1 v/v) and dissolved in 200 μl MS grade water. Further, extract was passed through Sep-Pak column (Waters, Ireland), eluted in 50 μl of MS grade water containing 0.1% formic acid and analyzed for the FB1 detection using LC-MS. 

### Detection of fumonisin B1 (FB1) by liquid chromatography with tandem mass spectrometry (LC-MS-MS).

An acuity UPLC system (Waters, USA) was used to perform reverse-phase chromatographic partition of the FB1. Peptide BEH C18 column was used for separation with dimensions 2.1 mm x 150 mm, 1.7 μm particle size, and 4 μL injection volume. The column temperature was set at 40 ºC, and 8 ºC was the temperature of the sample manager. The separation was carried out with formic acid (0.1%) and water (A); and formic acid (0.1%) and acetonitrile (B) as a mobile phase. An initial gradient used was 90% A and 10% B for 3 min, and then solvent B was increased linearly to 90% within 10 min and was kept constant for 2 min. Further, solvent B was decreased linearly to 10% in 3 min. To avoid carryover in the next acquisition, the column was washed between two acquisitions. The flow rate was 0.5 ml min^-1^. Eluents were acquired in Mass Spectrometer (TSQ Quantum Access Max mass spectrometer-Thermo Scientific) in positive electrospray ionization (ESI) mode. Each sample was acquired in triplicates. In the ESI+ mode, the MS spray voltage was 4.2 kV. The capillary temperature was 300 ºC and probe heating temperature 320 ºC with the sheath gas at 45 arbitrary units and the aux gas was 12 arbitrary units. 

### Genetic diversity of *F. verticillioides* using Inter Simple Sequence Repeat (ISSR)

The high-frequency occurring *F*. *verticillioides* isolates were further processed to study the genetic diversity using ISSR markers. The primers were selected based on the polymorphic and reproducible banding patterns to characterize all the *F*. *verticillioides* isolates; the 20 different ISSR primers containing di or tri-nucleotide repeats were used in the present study (Table S1). PCR was carried out in a reaction volume of 20 μL. The amplification mixture consisted of 1 μL of each forward and reverse primer (10 µmol L-1), 10 μL Taq master mix, 8 μL of MilliQ water, and 40 ng template DNA. Sterile MilliQ water was used as a negative control for each experiment. The PCR program was set as an initial denaturation at 95 ºC for 5 min, followed by 35 cycles of 95 ºC for 30 sec, 44 ºC for 45 sec, and 72 ºC for 2 min with a final extension at 72 °C for 7 min. The PCR-amplified products were loaded onto 20g L−1 agarose gel electrophoresis in 1x TAE buffer and visualized under a UV trans-illuminator gel documentation system. The ISSR fingerprint data was analyzed based on the presence or absence of a particular allele indicated as 1 and 0, respectively. The pairwise distance among the strains was calculated using Jaccard’s coefficient from the binary matrix. The distance matrices were used to cluster the strains by Unweighted Pair Group Method with arithmetic means (UPGMA). To identify the best threshold microsatellite partitions, the Adjusted Rand value was calculated for each UPGMA/Dice dendrograms to compare each possible combination of microsatellite regions.

### Data analysis

Annotation of sequenced TEF1-α gene was performed using BlastN with default parameters against the NR database. The blast hit results were filtered based on Maximum Query Coverage with maximum Identity and lowest e-value. Preference was given to *F*. *verticillioides* TEF1-α in cases where there was an identical hit from other strains. Phylogenetic analysis was done by performing ClustalW of the TEF1-α gene sequences from the isolates. Results of Clustal W were used as input to construct various trees to identify non-*F*. *verticillioides* gene clusters. The outliers were removed from the analysis, and distance-based trees were constructed. Interactive Tree of Life online tool was used to construct and visualize trees with Newick format as input for analysis. For ISSR analysis, the presence or absence of amplicon observed at a particular locus was scored as 1 and 0 correspondingly, and pairwise distance among the strains was calculated. Amplicon data was grouped into a 500bp window and provided input to unsupervised hierarchical clustering using the UPGMA method. The resultant file was used to identify Clades based on the distance score. Clustering and visualization were done using MegaX software. The tree file was used as input to the ITOL (Interactive Tree Of Life) online tool and the metadata (Geographical and Virulence) to perform circular tree visualization of the clades.

## Results

### Isolation and molecular identification of *Fusarium* species

**Figure 1 -:**
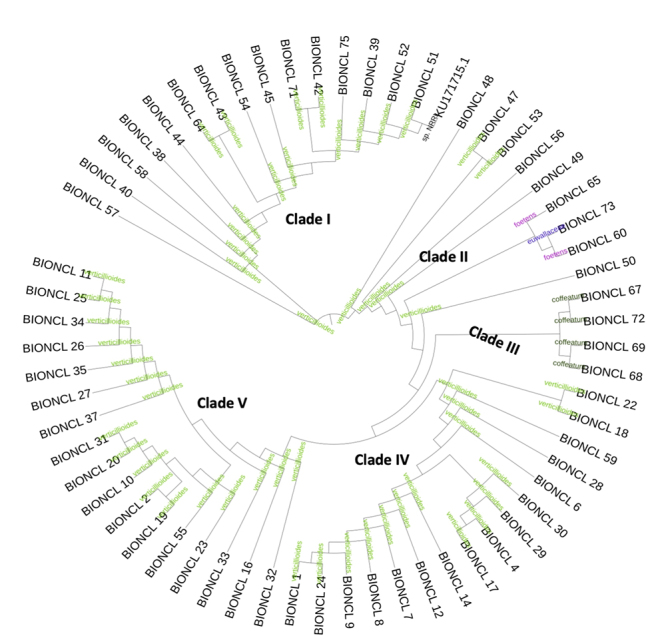
Phylogenetic analysis of *Fusarium* strains based on TEF1-α gene sequences.

**Figure 2 -:**
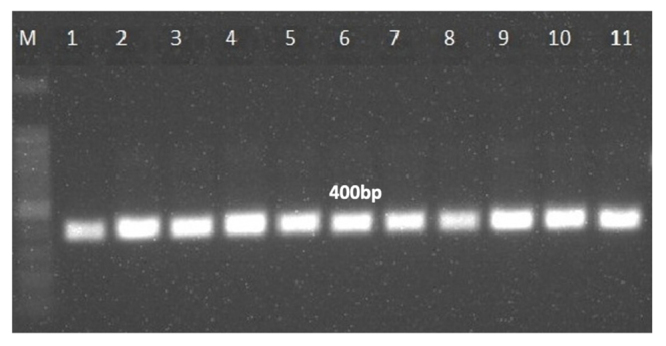
*Fusarium verticillioides* species specific PCR assay.

A total of 140 maize samples were collected from different geographical regions of India and processed for isolation of *Fusarium* species to ensure the grain quality. A total of 61 different *Fusarium* isolates were recovered from maize grain samples. The isolated colonies were morphologically differentiated based on the colony characteristics accompanied by microscopic identification of microconidia and macroconidia. Further, molecular identification of *Fusarium* species was performed using RNA transcript of TEF1-α gene sequences which resulted a single and specific amplicon at 300 bp (Figure S1). The TEF1-α gene sequences were subjected to BLAST analysis using the NCBI database and analyzed for the species-level identification using query coverage, percent identity, and sequence similarity greater than 99%. The molecular evolutionary lineages and phylogenetic tree was constructed using MEGA software version X. Amongst, *F*. *verticillioides* (88.52%) was the most dominating species, followed by *F*. *coffeatum* (6.5%), *F*. *foetens* (3.27%), and *F*. *euwallaceae* (1.63%), were recovered from the Indian stored maize samples. It is interesting to note that, we first time report *F*. *coffeatum*, *F*. *foetens*, and *F*. *euwallaceae* from maize grain in India. However, based on phylogenetic analysis, *Fusarium* strains were grouped into five major clades. Clade I contained *F*. *verticillioides* 15 strains, clade II is composed of 9 strains with different species, Clade III contained 4 *F*. *coffeatum* strains, clade IV, contained 16, and clade V consists of the highest 17 strains of *F*. *verticillioides* (Figure 1). Moreover, *F*. *coffeatum*, *F*. *foetens*, and *F*. *euwallaceae* were grouped in between the clade II and III, suggesting closeness of these species (more similar). The TEF1-α gene sequences showed more than 90% sequence similarity and were perfectly aligned into the clades and sub-clades. This demonstrated that *Fusarium* species have a wide range of diversity within the same species collected from the single host. Furthermore, *F*. *verticillioides* strains were evaluated using species-specific primers showed the target single amplicon of 400 bp specific to *F*. *verticillioides* and confirmed to the species specificity (Figure 2).

### Molecular detection and FB1 production 

The early detection of mycotoxigenic fungi in food and feed is of paramount importance. In this investigation, isolated *Fusarium* strains were processed for detection of FUM1 and FUM13 genes involved in FB1 biosynthesis. The FUM1 and FUM13 gene-specific amplification were observed at 927 bp and 852 bp, respectively (Figures S2, S3). Most of the *Fusarium* isolates had shown positive for FB1gene. Furthermore, FB1 production was confirmed by HPLC/LC-MS (Figure S4). About 90% of the strains are FB1 producers confirmed by both molecular and chromatographic analysis (Table S2). The MRM test was utilized to identify FB1 from *Fusarium* isolates, and the transitions used for FB1 are m/z 722.4 to m/z 334.2/352.2.

### Effect of *Fusarium* on maize growth inhibition

*In-vitro* growth inhibition studies results showed a negative effect on maize seed germination, and root and shoot growth elongation inhibition. The results showed 9, 22, and 26 showed high, moderate, and low inhibition of growth rates (11 and 24%), respectively. Interestingly, four isolates of *Fusarium* species have not displayed any effect on the maize, either on seed germination or shoot and root growth when compared to untreated control. The highest growth inhibition shown by nine isolates was collected from MH, AP, RJ, JK, and MP, and the four isolates that were not affected growth were from MP, RJ, and the UK. The rest of the isolates obtained from various states exhibited low to moderate inhibition of growth and development. *F*. *verticillioides*, *F*. *coffeatum*, *F*. *foetens*, and *F*. *euwallaceae* showed variations in their growth inhibition patterns (Table 1). However, more detailed studies are also needed for the confirmation of pathogenicity with other maize genotypes. 

**Table 1 -:** *In-vitro* effect of Fumonisin B1 producing *Fusarium verticillioides* on growth of maize grains.

Isolates code	Fusarium species	Seed germination inhibition (%)	Root inhibition (%)	Shoot inhibition (%)
BIONCL-1	*F*.*verticillioides*	40	8.55	8.24
BIONCL-2	*F*.*verticillioides*	40	19.81	28.35
BIONCL-4	*F*.*verticillioides*	30	19.81	5.67
BIONCL-6	*F*.*verticillioides*	60	4.05	19.58
BIONCL-7	*F*.*verticillioides*	10	16.66	21.13
BIONCL-8	*F*.*verticillioides*	70	10.81	8.76
BIONCL-9	*F*.*verticillioides*	20	11.71	1.28
BIONCL-10	*F*.*verticillioides*	80	12.16	7.41
BIONCL-11	*F*.*verticillioides*	30	9.00	6.28
BIONCL-12	*F*.*verticillioides*	20	6.75	7.21
BIONCL-14	*F*.*verticillioides*	20	40.09	32.98
BIONCL-16	*F*.*verticillioides*	40	23.42	21.13
BIONCL-17	*F*.*verticillioides*	70	47.29	46.39
BIONCL-18	*F*.*verticillioides*	70	35.58	20.61
BIONCL-19	*F*.*verticillioides*	90	25.67	28.35
BIONCL-20	*F*.*verticillioides*	40	25.67	28.35
BIONCL-22	*F*.*verticillioides*	60	7.20	9.02
BIONCL-23	*F*.*verticillioides*	70	20.04	21.90
BIONCL-24	*F*.*verticillioides*	90	2.25	15.46
BIONCL-25	*F*.*verticillioides*	70	8.10	1.54
BIONCL-26	*F*.*verticillioides*	90	2.70	3.09
BIONCL-27	*F*.*verticillioides*	70	13.96	17.52
BIONCL-28	*F*.*verticillioides*	60	4.95	1.54
BIONCL-29	*F*.*verticillioides*	20	24.77	21.13
BIONCL-30	*F*.*verticillioides*	70	22.07	19.58
BIONCL-31	*F*.*verticillioides*	80	50.90	30.41
BIONCL-32	*F*.*verticillioides*	40	42.34	35.56
BIONCL-33	*F*.*verticillioides*	00	20.72	11.81
BIONCL-34	*F*.*verticillioides*	90	0.00	21.64
BIONCL-35	*F*.*verticillioides*	80	21.59	17.11
BIONCL-37	*F*.*verticillioides*	60	65.31	56.70
BIONCL-38	*F*.*verticillioides*	80	30.63	37.62
BIONCL-39	*F*.*verticillioides*	90	16.66	38.14
BIONCL-40	*F*.*verticillioides*	50	67.11	59.79
BIONCL-42	*F*.*verticillioides*	80	72.07	62.82
BIONCL-43	*F*.*verticillioides*	90	32.8	36.08
BIONCL-44	*F*.*verticillioides*	60	34.23	32.98
BIONCL-45	*F*.*verticillioides*	40	43.24	39.17
BIONCL-47	*F*.*verticillioides*	90	15.76	28.35
BIONCL-48	*F*.*verticillioides*	20	25.67	39.17
BIONCL-49	*F*.*verticillioides*	90	82.43	71.64
BIONCL-50	*F*.*verticillioides*	90	12.61	26.80
BIONCL-51	*F*.*verticillioides*	10	11.10	27.03
BIONCL-52	*F*.*verticillioides*	70	8.56	11.10
BIONCL-53	*F*.*verticillioides*	NA	NA	NA
BIONCL-54	*F*.*verticillioides*	00	26.57	14.94
BIONCL-55	*F*.*verticillioides*	NA	NA	NA
BIONCL-56	*F*.*verticillioides*	50	6.78	28.04
BIONCL-57	*F*.*verticillioides*	30	NA	NA
BIONCL-58	*F*.*verticillioides*	60	NA	NA
BIONCL-59	*F*.*verticillioides*	70	7.02	31.07
BIONCL-64	*F*.*verticillioides*	10	7.20	18.55
BIONCL-71	*F*.*verticillioides*	80	4.95	5.67
BIONCL-75	*F*.*verticillioides*	80	28.37	38.14
BIONCL-60	*F*. *foetens*	NA	66.21	56.70
BIONCL-65	*F*. *foetens*	80	26.12	19.07
BIONCL-67	*F*. *coffeatum*	60	19.81	15.97
BIONCL-68	*F*. *coffeatum*	80	60.81	49.48
BIONCL-69	*F*. *coffeatum*	70	71.17	74.22
BIONCL-72	*F*. *coffeatum*	60	13.51	40.20
BIONCL-73	*F*. *euwallaceae*	90	41.93	56.49
	Control	NA	NA	NA

### ISSR fingerprint

**Figure 3 -:**
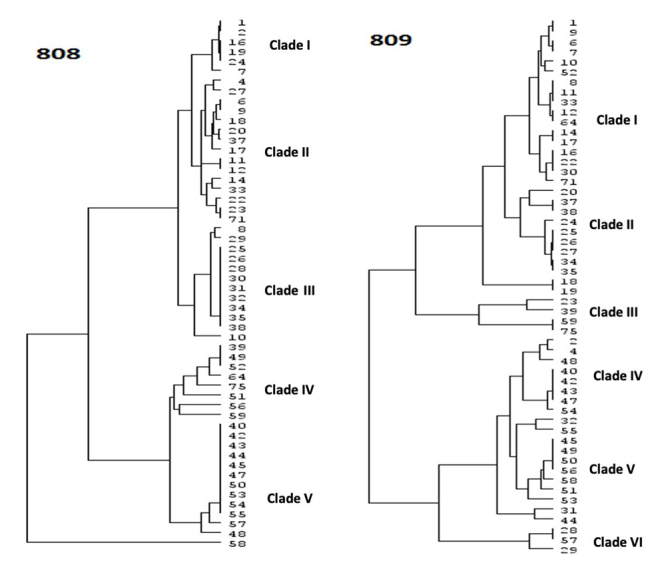
Phylogenetic analysis of *F*. *verticillioides* strains by ISSR fingerprint (AG)_8_C (808) and (AG)_8_G (809).

The genetic diversity of *F*. *verticillioides* was determined using microsatellite ISSR fingerprints. The polymorphic nature of all *F*. *verticillioidies* strains was representative of each of the ten states studied in India. Twenty ISSR primers were used for this study, however, only two ISSR primers (AG)8C and (AG)8G showed 99-100% reproducibility of banding patterns for *F*. *verticillioides*. The data with other primers were non-reproducible and less polymorphic, which led to their omission from the present study (data not shown). The primers (AG)8C produced banding patterns ranged 1-14 bands within 300-6kb and (AG)8G displayed 2-10 bands within 350-3kb amplicons (Figures S5, S6). The dendrograms obtained using Unweighted Pair Group with Arithmetic Mean (UPGMA/Dice) with (AG)8C primer fingerprints form five major clades (I to V) and 10 to 12 sub-clades, whereas (AG)8G primer finger-prints produced six major clades (I to VI) and 10 to 11 sub-clades that represents the high diversity among the *F*. *verticilliodies* strains which is randomly distributed and not withstand with the geographic regions (Figure 3).

**Figure 4 -:**
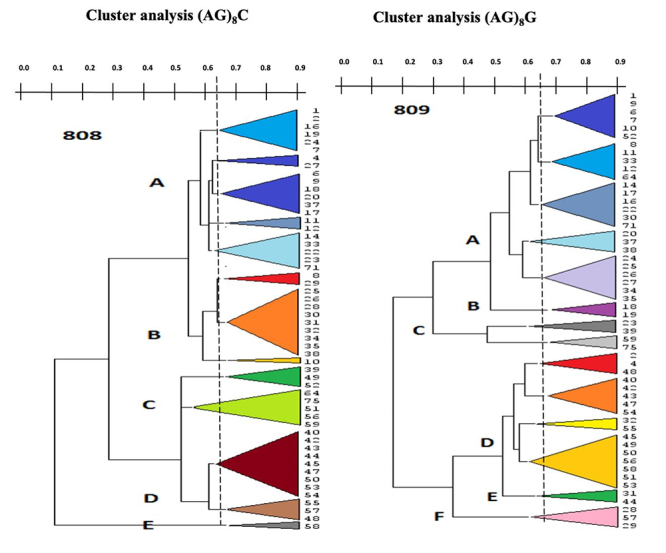
Diversity and comparative partition using cluster analysis.

Furthermore, the statistical discriminatory power of both primers was tested using Simpson’s (SID) and Shannon’s (H) indices for measuring diversity by comparing the partitions online tool. SID was used to calculate the discriminatory potential of the various sets of primers from typing systems, and it indicated the probability of the different population data sets. The SID index increases the discriminatory capacity of the typing technique. The confidence interval (CI) can be compared with the original Non-Approximated Confidence Interval (CINA). The SID and H indices data obtained after analysis are shown in Table 2. At 95% CI, SID and entropy H were resulted equivalent for both the primers. Furthermore, (AG)8G fingerprint specifies the microsatellite component better than those with the (AG)8 C fingerprint. The partition computations for each UPGMA/Dice dendrogram of the microsatellite component were adjusted from 50 to 95 percent. For both (AG)8G and (AG)8C, the maximum coefficient value was determined to be 0.230 at a threshold of 65 percent (Figure 4).SID partitions value reported as 0.762 with (AG)8C at this threshold, 13 clusters found with SID values at these cutoffs. Similarly, (AG)8G created 14 clusters with SID values of 0.785 for the partitions, and there was no significant difference between (AG)8G and (AG)8C, except for BIONCL-20, BIONCL-37, BIONCL-28, BIONCL-29, BIONCL-40, BIONCL-42, BIONCL-43, BIONCL-47, BIONCL-51, and BIONCL-56, which were classified into distinct clades. In both ISSR typing and *F*. *verticilliodes* were found to target separate loci. 

**Table 2 -:** Diversity study of ISSR finger print with Simpson’s index of diversity (SID) and Jackknife pseudo-values.

ISSR primer	# partitions	Simpson’s ID	Jackknife pseudo-values CI (95%)	H
(AG)_8_C	2	0.230	(0.088-0.372)	0.556
	2	0.283	(0.141-0.425)	0.650
	4	0.663	(0.593-0.733)	1.666
	4	0.760	(0.741-0.778)	1.987
	6	0.762	(0.700-0.824)	2.201
(AG)_8_G	2	0.073	(-0.027-0.172)	0.229
	4	0.595	(0.489-0.702)	1.492
	3	0.579	(0.492-0.665)	1.352
	5	0.755	(0.705-0.806)	2.078
	7	0.785	(0.729-0.842)	2.364

**Figure 5 -:**
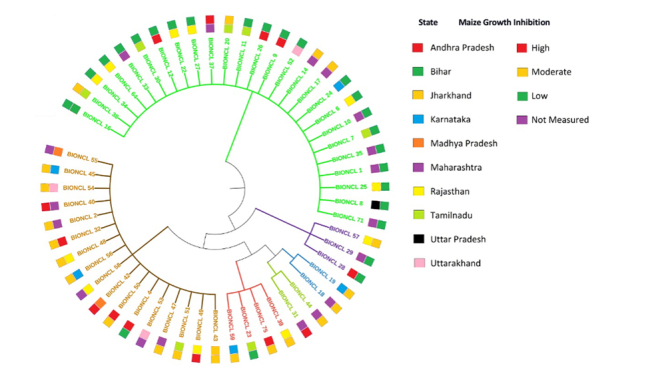
Phylogenetic analysis of *Fusarium verticillioides* strains based on (AG)8G ISSR fingerprint and their growth inhibitory pathogenicity distribution in maize.

The UPGMA dendrogram of (AG)8G fingerprint were more distinct than the (AG)8G primer was examined based on major clades and a total number of sub-clades observed. However, the phylogenetic tree was constructed using (AG)8G ISSR primer. It forms six major clades in which distributed strains notwithstanding their geographic origin, but interestingly, proposed clades significantly correlate with the growth inhibition pattern. Clade I contained more than 75% strains that showed low inhibition of growth except four strains originated from TN, and MH showed moderate or high growth inhibition, clade II strains showed only moderate growth inhibition, whereas clades (III, IV, V) strains showed moderate or high growth inhibition except BIONCL 4 strain demonstrated low disease severity and three strains have not shown any effect on growth and development of maize. However, clade VI strains demonstrated low growth inhibition, except BIONCL 57 showed no growth inhibition (Figure 5). More comprehensive research, may necessitate investigating the genetic/molecular diversity of the same species in maize genotypes.

## Discussion

*Fusarium* species are highly variable in their morphological and evolutionary characteristics. Classification of *Fusarium* based on morphological observations revealed more than 1,000 species, strains, and forms (Crous et al., 2021). There is extensive diversity among the unresolved *Fusarium* within the species (Devi et al., 2016). A detailed and reliable classification system is needed because of its pathogenicity to the plants and humans. In the present study, the molecular identification of *Fusarium* species was performed using TEF1-α as a highly desired protein-coding marker. Most of the classifications within species were based on the pathogenic behaviour and vegetative compatibility groups (VGC) that aided inaccurate identification of TEF1-α gene (Geiser et al., 2004). It is a diverse genus with various species complexes including FGSC, GFSC, FOSC, FSSC, FDSC, and FIESC associated with and infected with different food crops (Thomas et al., 2019, Crous *et al*., 2021). Diverse *Fusarium* species were recovered from maize samples collected from ten states of India. This led to the recovery of a total of 61 isolates, of which 54 were *F*. *verticillioides* and are the most dominating species, followed by *F*. *coffeatum*, *F*. *foetens*, and *F*. *euwallaceae*. *F*. *verticillioides* (Nagaraja et al., 2016), with the characteristic feature of producing a variety of biothreat-mycotoxins (Mohammed et al., 2016). Unfortunately, no such extensive studies were available on molecular diversity of species of *Fusarium* in India. The prevalence of *F*. *verticillioides* reported for contamination of different crops such as paddy, sorghum, maize, small cereal grains, poultry, and cattle feed (Navale et al., 2022c). *F*. *verticillioides* strains (74%) were associated with various food samples from Karnataka, India, in which 42% of the strains showed positive signals for FUM production (Nagaraj and Sreenivasa 2017). *F*. *verticillioides* reported causing stalk corn rot in maize cultivars in Assam, India (Borah et al., 2016) and even resistant maize cultivars (Ridenour et al., 2016).

*Fusarium verticillioides* has been considered a widely spreading plant pathogen, and produces excess FUM production (Shin et al., 2014). It belongs to the FFSC and is known to cause head, stalk, and root rots in maize due to the active production of FUM (Shin *et al*., 2014). *Fusarium coffeatum* is a member of the *Fusarium incarnatum-equiseti* species complex (FIESC), whereas *F*. *foetens* is a member of the *Fusarium oxysporum* species complex (FOSC) and a well-known plant pathogen that infects a wide range of cereals, including maize and ornamental crops (Xia et al., 2019). It belongs to the *Fusarium solani* species complex (FSSC), and was identified in an invasive ambrosia beetle from Israel and California, FSSC, showing the distinctive ecologies and exclusive lineage within Clade 3 of the FSSC (Freeman et al., 2013). In Indian maize, there were no reports available for these species of plant-pathogen with toxin production (Summerell et al., 2019). To the best of our knowledge, there were no reports available on *F*. *coffeatum*, *F*. *euwallaceae*, or *F*. *foetens* associated with food grain samples from India. In the present study, for the first time, we reported these species from stored Indian maize samples. 

In addition to the precise identification of the species, molecular detection of mycotoxins biosynthetic genes are of utmost importance for evaluating the toxigenic fungi and their potent toxins, which are necessary to ensure food quality. Molecular methods have become a robust alternative due to their flexibility, precision, and low time requirements compared to conventional methods (Raja et al., 2017). The developed PCR assay was able to detect FB1 in the strains isolated from maize grain samples due to the sensitivity and specificity of the primers. 

In this investigation, we isolated 54 *F*. *verticillioides* strains and evaluated their genetic diversity using two ISSR fingerprints (Vieira et al., 2016). The ISSR primers recognized five to six clades and about 50% of the diversity on ISSR fingerprints. The microsatellite DNA formed several repetitive motifs that demonstrated substantial distinction among Indian maize isolates. Moreover, no correlation was observed between the clustering within *F*. *verticillioides* and geographical regions. Nevertheless, (AG)8G primer-based fingerprints formed six major clades, which showed a strong correlation with maize growth inhibition. The obtained clades were distributed with the disease severity, demonstrating the variation in pathogenicity within the *F*. *verticillioides* (Nayaka *et al*., 2011). Interestingly, Leyva-Madrigal et al. (2014) studied the polymorphic nature of ISSR markers and 470 microsatellites in the genome of *F*. *verticillioides*. ISSR fingerprints facilitated the studies on the relationships between *F*. *verticillioides* and showed SID ranged from 0.034 to 0.78 with both the primers. The (AG)8C and (AG)8G markers revealed 252 and 368 microsatellites sites, respectively, in the genome of *F*. *verticillioides*. *Fusarium verticillioides* enabled the production of FB1 in strains. The phylogenetic analysis of *F*. *verticillioides* sequences resulted in five different clades that demonstrated the varying degree of diversity within the species. In the recent past, the biodiversity in *Fusarium* led to the description of several new specific and infra-specific taxa by examining the large populations. Associative mapping using diverse genotypes was a promising and practical approach to gain valuable insights that determined the functional variability of both known and unknown genes (Irzykowska et al., 2012). The different *Fusarium* species were profiled for toxigenic potentials by HPLC/LC-MS and PCR for FB1 production. The data revealed that more than 90% strains are FB1 producers (Table S2). In addition, about 31 strains showed moderate to high inhibition, including seed germination, shoot, and root growth inhibition of the maize (Table 1). The FB1 and other toxins and pathogen proteins produced by *F*. *verticillioides* might be a virulence factor in disease development (Mclean, 1995). In addition, these species were known to possess virulent genes, which translated to the pathogenic proteins, thereby causing plant pathogenesis. However, there is a need to prove this hypothesis through mechanistic studies, determining the exact pathogenicity factors (Tripathi and Misra, 1983).

## Conclusion

In the present study, we isolated toxigenic *F*. *verticillioides*, which is a major contaminant of stored maize grains in India. For the first time, new reports of the species *F*. *coffeatum, F*. *euwallaceae,* and *F*. *foetens* from maize grain, India, which is capable of producing FB1. The polymorphic nature of *F*. *verticilliodes* exhibited SID and H indexes in the range of 0.78 and 2.36, respectively. The (AG) 8G ISSR fingerprint not only differentiates pathogenic populations in different species; but also segregates the pathogenic and non-pathogenic clades in *F*. *verticillioides* irrespective. The identified and characterized molecular markers are imperative for developing a disease-resistant crop breeding program, leading to effective control of the *Fusarium* pathogen. These findings might help to understand the molecular mechanisms of host-pathogen interaction and the virulence potential of the pathogen to propose disease management policies based on molecular markers. 

## Supplementary material

The following online material is available for this article:

Table S1 - Primers used in present study for molecular identification, mycotoxin detection and diversity study.

Table S2 - Molecular and analytical based detection of fumonisins (FUM) produced by *Fusarium* species.

Figure S1 - Molecular identification of *Fusarium* species by targeting TEF1-α gene.

Figure S2 - Molecular detection of Fumonisin by targeting Fum1 gene.

Figure S3 - Molecular detection of Fumonisins by FUM13 gene.

Figure S4 - LC-MS analysis of fumonisins B1 (FB1).

Figure S5 - PCR amplification of *Fusarium* isolates by (AG)8C ISSR primer.

Figure S6 - PCR amplification of *Fusarium* isolates by (AG)8G ISSR primer.
