# Supplementary material for: Genetic diversity of toxigenic Fusarium verticillioides associated with maize grains, India
Source: Genet Mol Biol. 2023 Apr 7;46(1):e20220073. doi: 10.1590/1678-4685-GMB-2022-0073 (PMC10084715; doi:10.1590/1678-4685-GMB-2022-0073)
Supplement: Table S1 - [file 1415-4757-GMB-46-1-e20220073-s1.pdf]

Genetics and Molecular Biology

https://doi.org/10.1590/1678-4685-GMB-2022-0073

Supplementary Material to "Genetic diversity of toxigenic Fusarium verticillioides associated with Maize Grains, India"

Table S1 - Primers used in present study for molecular identification, mycotoxin detection and diversity study.

Primer name	Primer sequence (5' – 3')	Gene targeted	Tm (˚C)	Amplicon size(bp)	Reference
EF1 F	ATGGGTAAGGARGACAAGAC	THF1-α	58	300	O'Donnell et al (1998)
EF2 R	GGARGTACCAGTSATCATGTT				
Vert f 1	GCGGGAATTCAAAAGTGGCC		59	400	Patino et al (2004)
Vert f 2	GAGGGCGCGAAACGGATCGG				
Fum1F	GAATCACCCTCAAGCACCAC	FUM1	58	927	Present study
Fum1R	CTTCATGGCTCTCAGAGCTTG				
Fum13 F	CTATGCTGTGCTTGTCAAGACTC	FUM13	57	852	Present study
Fum13 R	CTCCTGACAATCTCTCCGATCTC				
ISSR-1	CACACACACACAGT- (CA)6GT	ISSR	45	-	-
ISSR-2	CTCTCTCTCTCTCTCTTC - (CT)9TC	ISSR	50	-	-
ISSR-3	CACCACCACGC -(CAC)4GC	ISSR	54	-	-

Primer name	Primer sequence (5' – 3')	Gene targeted	Tm (˚C)	Amplicon size(bp)	Reference
ISSR-4	CACACACACACACACAA- (CA)9A	ISSR	47	-	-
ISSR-5	CACACACACACAAG -(CA)7AG	ISSR	48	-	-
ISSR-6	GAGAGAGAGAGAGG -(GA)7GG	ISSR	46	-	-
ISSR-7	CTCTCTCTCTCTCTCTGC - (CT)8GC	ISSR	50	-	-
ISSR-8	GTGGTGGTGGTGGTG - (GTG)6	ISSR	59	-	-
ISSR-9	GAGAGAGAGAGAGAGAGAC- (GA)10C	ISSR	52	-	-
ISSR-10	GAGAGAGAGAGAGAGAGAT- (GA)10T	ISSR	51	-	-
UBC808	GAGAGAGAGAGAGAGC -(AG)8C	ISSR	47	-	-
UBC809	AGAGAGAGAGAG AGA GG - (AG)8G	ISSR	48	-	-
UBC811	GAGAGAGAGAGAGAGAC - (GA)8C	ISSR	47	-	-
UBC813	CTCTCTCTCTCTCTCTT- (CT)8T	ISSR	46	-	-
UBC815	CTCTCTCTCTCTCTCTG - (CT)8G	ISSR	47	-	-
UBC816	CACACACACACA CACAT - (CA)8T	ISSR	47	-	-
UBC818	CACACACACACACACAG - (CA)8G	ISSR	51	-	-
UBC819	GTCTGTGTGTGTGTGTA - (GT)8A	ISSR	49	-	-
UBC825	ACACACACACACACACT- (AC)8T	ISSR	51	-	-

Primer name	Primer sequence (5’ – 3’)	Gene targeted	Tm (˚C)	Amplicon size(bp)	Reference
UBC864	ATGATGATGATGATGATG - (ATG)6	ISSR	44	-	-
