# Supplementary material for: Genetic diversity of toxigenic Fusarium verticillioides associated with maize grains, India
Source: Genet Mol Biol. 2023 Apr 7;46(1):e20220073. doi: 10.1590/1678-4685-GMB-2022-0073 (PMC10084715; doi:10.1590/1678-4685-GMB-2022-0073)
Supplement: Table S2 - [file 1415-4757-GMB-46-1-e20220073-s2.pdf]

Genetics and Molecular Biology
https://doi.org/10.1590/1678-4685-GMB-2022-0073

Supplementary Material to "Genetic diversity of toxigenic Fusarium verticillioides associated with Maize Grains, India"

Table S2 - Molecular and analytical based detection of fumonisins (FUM) produced by *Fusarium* species.

Isolates code	*Fusarium* spp.	Source	PCR FUM1	PCR FUM13	LC-MS FUM
BIONCL-1	*F.verticillioides*	Sillod, Maharashtra	+	+	+
BIONCL-2	*F.verticillioides*	Buldhana, Maharashtra	+	+	+
BIONCL-4	*F.verticillioides*	Warangal, Andhra Pradesh	+	+	+
BIONCL-6	*F.verticillioides*	Jodhpur, Rajasthan	+	+	+
BIONCL-7	*F.verticillioides*	Coimbatore, Tamilnadu	+	+	+
BIONCL-8	*F.verticillioides*	Lucknow, Uttar Pradesh	+	+	+
BIONCL-9	*F.verticillioides*	Vijayawada, Andhra Pradesh	+	+	+
BIONCL-10	*F.verticillioides*	Sillod, Maharashtra	+	+	+
BIONCL-11	*F.verticillioides*	Coimbatore, Tamilnadu	+	+	+
BIONCL-12	*F.verticillioides*	Vijayawada, Andhra Pradesh	+	+	+
BIONCL-14	*F.verticillioides*	Pune, Maharashtra	+	+	+
BIONCL-16	*F.verticillioides*	Patna, Bihar	+	+	+
BIONCL-17	*F.verticillioides*	Beed, Maharashtra	+	+	+
BIONCL-18	*F.verticillioides*	Aurangabad, Maharashtra	+	+	+
BIONCL-19	*F.verticillioides*	Shimoga, Karnataka	+	+	+
BIONCL-20	*F.verticillioides*	Coimbatore, Tamilnadu	+	+	+
BIONCL-22	*F.verticillioides*	Udaipur, Rajasthan	-	-	-
BIONCL-23	*F.verticillioides*	Coimbatore, Tamilnadu	-	-	-
BIONCL-24	*F.verticillioides*	Bidar, Karnataka	+	+	+
BIONCL-25	*F.verticillioides*	Jaipur, Rajasthan	+	+	+
BIONCL-26	*F.verticillioides*	Khammam, Andhra Pradesh	-	-	-
BIONCL-27	*F.verticillioides*	Jodhpur, Rajasthan	+	+	+

Isolates code	*Fusarium* spp.	Source	PCR FUM1	PCR FUM13	LC-MS FUM
BIONCL-28	*F.verticillioides*	Khammam, Andhra Pradesh	+	+	+
BIONCL-29	*F.verticillioides*	Aurangabad, Maharashtra	+	+	+
BIONCL-30	*F.verticillioides*	Coimbatore, Tamilnadu	+	+	+
BIONCL-31	*F.verticillioides*	Jalna, Maharashtra	+	+	+
BIONCL-32	*F.verticillioides*	Khammam, Andhra Pradesh	+	+	+
BIONCL-33	*F.verticillioides*	Aurangabad, Maharashtra	+	+	+
BIONCL-34	*F.verticillioides*	Jaipur, Rajasthan	+	+	+
BIONCL-35	*F.verticillioides*	Sillod, Maharashtra	+	+	+
BIONCL-37	*F.verticillioides*	Sillod, Maharashtra	-	+	+
BIONCL-38	*F.verticillioides*	Coimbatore, Tamilnadu	+	+	+
BIONCL-39	*F.verticillioides*	Jaipur, Rajasthan	-	-	-
BIONCL-40	*F.verticillioides*	Jalgaon, Maharashtra	-	-	-
BIONCL-42	*F.verticillioides*	Indore, Madhya Pradesh	+	+	+
BIONCL-43	*F.verticillioides*	Ranchi, Jharkhand	+	+	+
BIONCL-44	*F.verticillioides*	Pune, Maharashtra	+	+	+
BIONCL-45	*F.verticillioides*	Gulbarga, Karnataka	+	+	+
BIONCL-47	*F.verticillioides*	Beed, Maharashtra	+	+	+
BIONCL-48	*F.verticillioides*	Udaipur, Rajasthan	+	+	+
BIONCL-49	*F.verticillioides*	Jodhpur, Rajasthan	+	+	+
BIONCL-50	*F.verticillioides*	Warangal, Andhra Pradesh	+	+	+
BIONCL-51	*F.verticillioides*	Coimbatore, Tamilnadu	+	+	+
BIONCL-52	*F.verticillioides*	Kotdwar, Uttarakhand	+	+	+
BIONCL-53	*F.verticillioides*	Pallavi, Uttarakhand	+	+	+
BIONCL-54	*F.verticillioides*	Rudraprayag, Uttarakhand	+	+	+
BIONCL-55	*F.verticillioides*	Indore, Madhya Pradesh	-	-	-
BIONCL-56	*F.verticillioides*	Vijaypura, Karnataka	+	+	+
BIONCL-57	*F.verticillioides*	Jodhpur, Rajasthan	+	+	+
BIONCL-58	*F.verticillioides*	Jaipur, Rajasthan	+	+	+

**Isolates code**	***Fusarium* spp.**	**Source**	**PCR FUM1**	**PCR FUM13**	**LC-MS FUM**
BIONCL-59	*F.verticillioides*	Bagalkot, Karnataka	+	+	+
BIONCL-64	*F.verticillioides*	Udaipur, Rajasthan	+	+	+
BIONCL-71	*F.verticillioides*	Beed, Maharashtra	+	+	+
BIONCL-75	*F.verticillioides*	Vijayawada, Andhra Pradesh	+	+	+
BIONCL-60	*F. foetens*	Warangal, Andhra Pradesh	+	+	+
BIONCL-65	*F. foetens*	Jodhpur, Rajasthan	+	+	+
BIONCL-67	*F. coffeatum*	Devanagri , Karnataka	+	+	+
BIONCL-68	*F. coffeatum*	Sillod, Maharashtra	+	+	+
BIONCL-69	*F. coffeatum*	Indore, Madhya Pradesh	+	+	+
BIONCL-72	*F. coffeatum*	Jalgaon, Maharashtra	+	+	+
BIONCL-73	*F. euwallaceae*	Ranchi, Jharkhand	+	+	+
