# Supplementary material for: Genetic diversity of toxigenic Fusarium verticillioides associated with maize grains, India
Source: Genet Mol Biol. 2023 Apr 7;46(1):e20220073. doi: 10.1590/1678-4685-GMB-2022-0073 (PMC10084715; doi:10.1590/1678-4685-GMB-2022-0073)
Supplement: Figure S4 - [file 1415-4757-GMB-46-1-e20220073-s6.pdf]

Genetics and Molecular Biology

https://doi.org/10.1590/1678-4685-GMB-2022-0073

Supplementary Material to "Genetic diversity of toxigenic Fusarium verticillioides associated with Maize Grains, India"

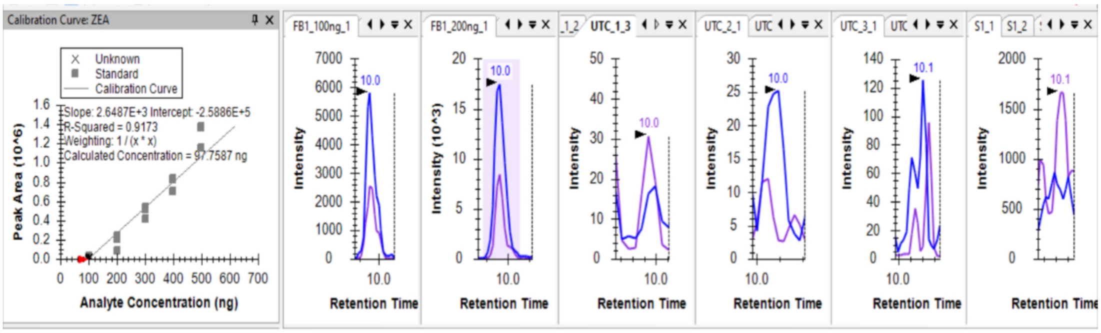

Figure S4 - LC-MS analysis of fumonisins B1 (FB1).
